# Supplementary material for: Paper-Based Humidity Sensor for Respiratory Monitoring
Source: Materials (Basel). 2022 Sep 16;15(18):6447. doi: 10.3390/ma15186447 (PMC9503997; doi:10.3390/ma15186447)
Supplement: Supplementary file 1 [file materials-15-06447-s001.zip › materials-1885739-supplementary.pdf]

Supporting Information

Paper-based humidity sensor for respiratory monitoring

Xiaoxiao Ma†, Shaoxing Zhang†, Peikai Zou, Ruya Li*, Yubo Fan*

1. Establishment of paper wettability model

1.1. The relationship between resistance of the sensor and water film thickness

According to the law of resistance,

$$R = \rho \frac{l}{S} \tag{S1}$$

where R is the resistance of the conductor, l and S are the conductor length and cross-sectional area in the direction of the current, and ρ is the resistivity of the conductor material. For PHS, the conductor is the paper fiber composite between the interdigital electrodes. The length of the PHS conductor is fork finger spacing, which is represented by d. The conductor cross-sectional area is that of the water layer condensates on the fiber in the composite. To calculate the cross-section area of the water layer, while printing paper can be seen as an isotropic fiber composite, the following equation is introduced:

$$A_f = \frac{1}{2\pi} V_f \tag{S2}$$

where A_f is the area fraction of the fiber sectional area on a paper section, and V_f is the volume fraction of the fiber, which can be calculated from known paper parameters and fiber density. By combining the above parameters and formulas, the relationship between resistance R and water layer thickness t is obtained,

$$R = \frac{\rho d r^2}{L h A_f ((r+t)^2 - r^2)} \tag{S3}$$

where d and L are the interfinger gap width and interfinger gap length of the interdigital electrode, h is the thickness of paper, r is the average radius of paper fiber, and A_f is the fiber

area fraction of the paper composite, and ρ is the resistivity of water.

1.2. The relationship between water film thickness and relative humidity

Water condensation theory was used to explain the condensation of vapor on hydrophilic paper fibers. Theoretically, four types of intermolecular forces, including hydration, electrostatic, van der Waals forces, and elastic strain, are taken into account [S1]. The disjoining pressure is introduced to evaluate the force, which can be calculated as

$$P(t)=\frac{Ue^{-t/\lambda}}{\lambda}(1+\frac{t}{\lambda}+\frac{\lambda^2}{t^2})+64k_BT\rho_\infty\gamma^2e^{-\kappa t}-\frac{A}{6\pi t^3}-\frac{E\varepsilon_0^2}{2}e^{-t/\lambda} \quad \text{(S4)}$$

and the meaning of each parameter will be explained later.

According to Israelachvili's theory of intermolecular and surface force in 1992, the disjoining pressure can also be written as [S2]

$$P(t)=-\frac{k_BT}{\upsilon}\ln\psi \quad \text{(S5)}$$

By combining the above two formulas, we can get the relationship between relative humidity and water layer thickness t on the fiber surface as

$$\psi=\exp\{-\alpha[\frac{Ue^{-t/\lambda}}{\lambda}(1+\frac{t}{\lambda}+\frac{\lambda^2}{t^2})+\beta e^{-\kappa t}-\delta\frac{1}{t^3}-\eta e^{-t/\lambda}]\} \quad \text{(S6)}$$

where ψ is the relative humidity, U is the free energy parameter in hydration energy, λ is the length scale parameter in hydration energy, and κ is the reciprocal of Debye length. $\alpha=\upsilon/k_BT$ is a constant related to the volume of a water molecule υ, Boltzmann constant k_B, and Kelvin temperature T. $\beta=64k_BT\rho_\infty\gamma^2$ is a constant related to k_B, T, the concentration of the solution far away from the surface ρ_∞ and γ is given by $\gamma=tanh(e\varphi_0/4k_BT)$. δ is in direct proportion to Hamaker constant A. $\eta=E\varepsilon_0^2/2$ is related to elastic modulus E and permittivity of vacuum ε_0.

2. Supplementary Table

Product or Literature	Physiological Parameter	Wearability	Distinguishing between oral and nasal breathing signals
PHS (this article)	Humidity	Stuck to skin	√
Sleep tracking pad [S3]	Body movement	Placed under mattress	×
Respiration monitor belt [S4]	Gas pressure	Pressurized and tied to the waist	×
Smart facemask [S5]	CO_2 gas concentration	Mask	×
Intelligent facemask [S6]	Airflow	Mask	×
Patch based on accelerator and gyroscope [S7]	Body movement	Stuck to skin	×
Ultrathin Flexible Sensor [S8]	Temperature	Inserted into nasal cavity	×
Smart Textile [S9]	Body movement	Tied to the chest	×

Supplementary Table S1. Comparing PHS with existing respiratory monitoring products and methods in literature.

3. Supplementary Figure

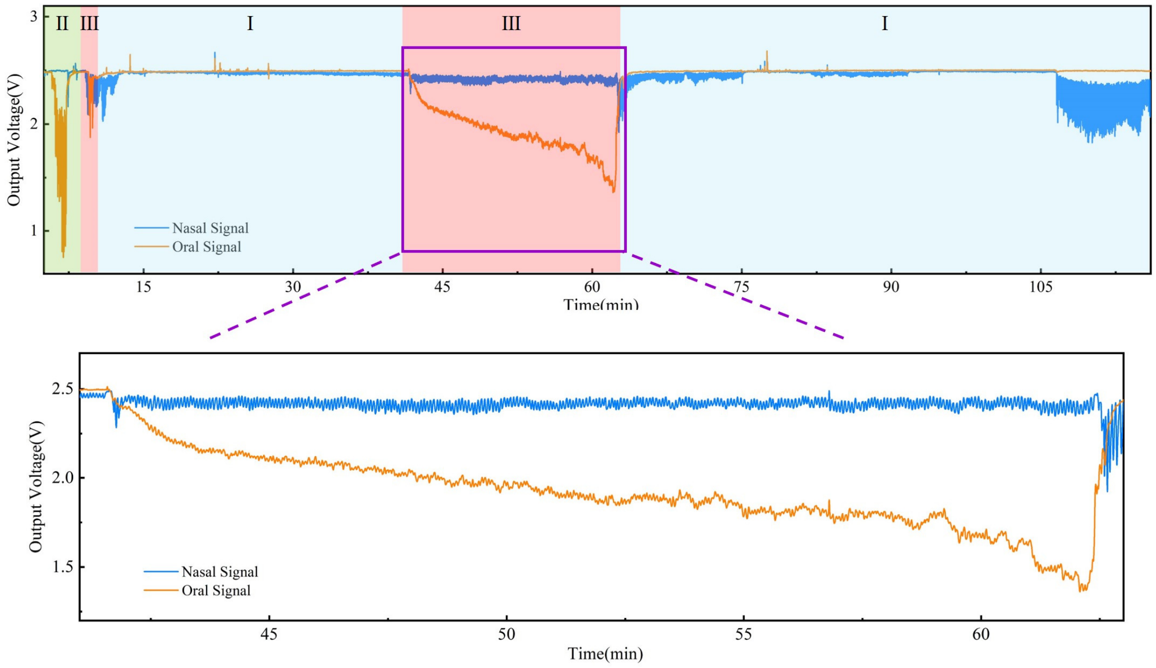

Supplementary Figure S1. Periodic and regular respiratory waveforms in both oral and nasal signals when the signal drifts. Overall signals of sleep test on healthy volunteer with local magnified image of the drift signal.

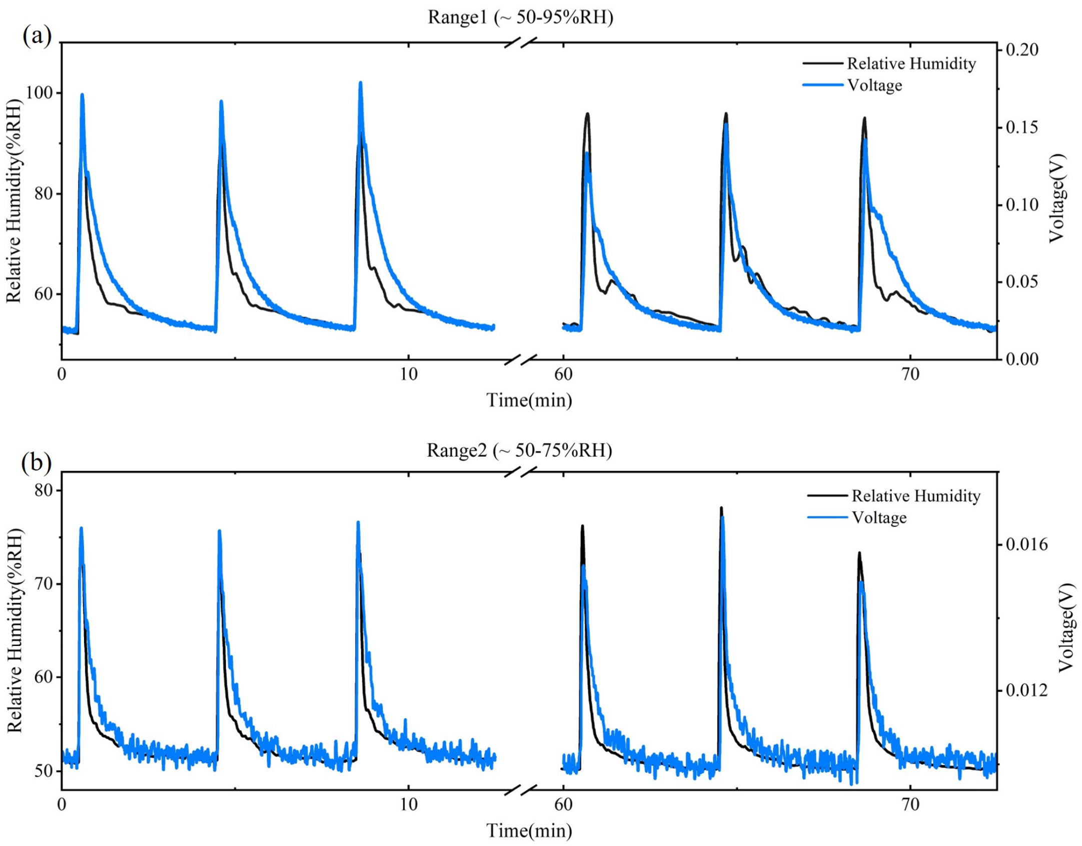

Supplementary Figure S2. Repeatability of 80gsm PHS. Sensor response to periodically varying relative humidity signals in different ranges of (a) ~ 50 - 95%RH and (b) ~ 50 - 75%RH.

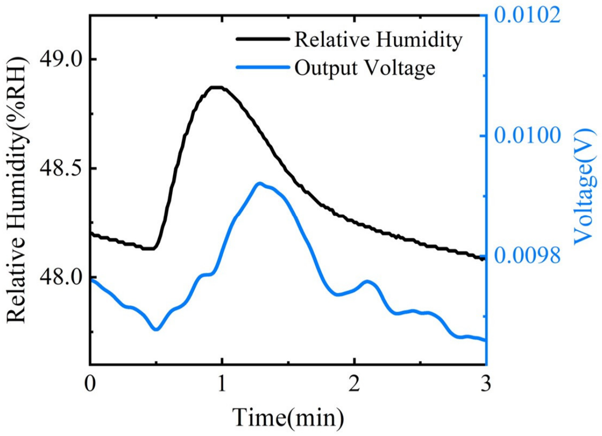

Supplementary Figure S3. Sensor gdetection limit experiment. Sensor's response to slight relative humidity changes of 0.74%RH.

References

[S1] Majumdar, A., and I. Mezic. "Instability of ultra-thin water films and the mechanism of droplet formation on hydrophilic surfaces." (1999): 964-971.

[S2] Israelachvili, Jacob N. Intermolecular and surface forces. Academic Press, 2011.

[S3] Edouard, Paul, et al. "Validation of the Withings Sleep Analyzer, an under-the-mattress device for the detection of moderate-severe sleep apnea syndrome." Journal of Clinical Sleep Medicine 17.6 (2021): 1217-1227.

[S4] Asadi, Ali, et al. "Inducing Changes in Breathing Patterns Using a Soft Robot." Proceedings of the 2022 ACM/IEEE International Conference on Human-Robot Interaction. 2022.

[S5] Escobedo, P., et al. "Smart facemask for wireless CO2 monitoring." Nature communications 13.1 (2022): 1-12.

[S6] Lu, Qixin, et al. "Intelligent facemask based on triboelectric nanogenerator for respiratory monitoring." Nano Energy 91 (2022): 106612.

[S7] Wang, Sikai, et al. "A new physiological signal acquisition patch designed with advanced respiration monitoring algorithm based on 3-axis accelerator and gyroscope." 2018 40th Annual International Conference of the IEEE Engineering in Medicine and Biology Society (EMBC). IEEE, 2018.

[S8] Zhang, Haodong, et al. "A Wearable Ultrathin Flexible Sensor Inserted Into Nasal Cavity for Precise Sleep Respiratory Monitoring." IEEE Transactions on Electron Devices 68.8 (2021): 4090-4097.

[S9] Massaroni, Carlo, et al. "Smart textile based on piezoresistive sensing elements for respiratory monitoring." IEEE Sensors Journal 19.17 (2019): 7718-7725.
